# {6,6′-Dibromo-4,4′-dichloro-2,2′-[*o*-phenyl­enebis(nitrilo­methyl­idyne)]diphenolato}nickel(II)

**DOI:** 10.1107/S1600536810010949

**Published:** 2010-03-27

**Authors:** Abdulaziz Ali, Norbani Abdullah, Mohd Jamil Maah, Kong Mun Lo

**Affiliations:** a Department of Chemistry, University of Malaya, 50603 Kuala Lumpur, Malaysia

## Abstract

In the title complex, [Ni(C_20_H_10_Br_2_Cl_2_N_2_O_2_)], the Ni^II^ ion is coordinated by two phen­oxy O atoms and two imino N atoms of the tetradentate ligand, forming a slightly distorted square-planar environment. The mol­ecule is essentially planar, with an r.m.s. deviation of 0.088 Å for the mean plane defined by all non-H atoms in the mol­ecule.

## Related literature

For applications of nickel(II) complexes containing nitro­gen and oxygen donor ligands, see: Chang *et al.* (2008 ▶). For related structures, see: Wang *et al.* (2003 ▶); Niu *et al.* (2009 ▶); Azevedo *et al.* (1994 ▶).
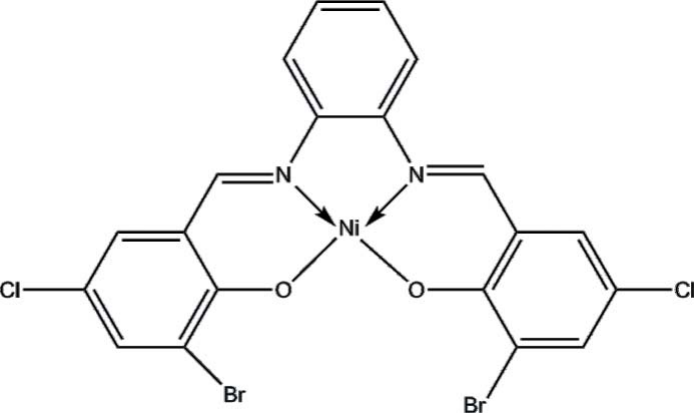


## Experimental

### 

#### Crystal data

[Ni(C_20_H_10_Br_2_Cl_2_N_2_O_2_)]
*M* *_r_* = 599.73
Monoclinic, 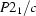

*a* = 10.4289 (2) Å
*b* = 9.2712 (2) Å
*c* = 20.6731 (4) Å
β = 102.101 (1)°
*V* = 1954.43 (7) Å^3^
*Z* = 4
Mo *K*α radiation
μ = 5.38 mm^−1^
*T* = 296 K
0.40 × 0.10 × 0.10 mm

#### Data collection

Bruker APEXII CCD area-detector diffractometer
Absorption correction: multi-scan (*SADABS*; Sheldrick, 1996 ▶) *T*_min_ = 0.222, *T*_max_ = 0.616
18381 measured reflections
4487 independent reflections
2921 reflections with *I* > 2σ(*I*)
*R*_int_ = 0.060

#### Refinement

*R*[*F* ^2^ > 2σ(*F* ^2^)] = 0.038
*wR*(*F* ^2^) = 0.085
*S* = 0.99
4487 reflections
302 parameters
H atoms treated by a mixture of independent and constrained refinement
Δρ_max_ = 0.62 e Å^−3^
Δρ_min_ = −0.46 e Å^−3^

### 

Data collection: *APEX2* (Bruker, 2008 ▶); cell refinement: *SAINT* (Bruker, 2008 ▶); data reduction: *SAINT*; program(s) used to solve structure: *SHELXS97* (Sheldrick, 2008 ▶); program(s) used to refine structure: *SHELXL97* (Sheldrick, 2008 ▶); molecular graphics: *X-SEED* (Barbour, 2001 ▶); software used to prepare material for publication: *publCIF* (Westrip, 2010 ▶).

## Supplementary Material

Crystal structure: contains datablocks I, global. DOI: 10.1107/S1600536810010949/lh5011sup1.cif

Structure factors: contains datablocks I. DOI: 10.1107/S1600536810010949/lh5011Isup2.hkl

Additional supplementary materials:  crystallographic information; 3D view; checkCIF report

## Figures and Tables

**Fig. 1.**
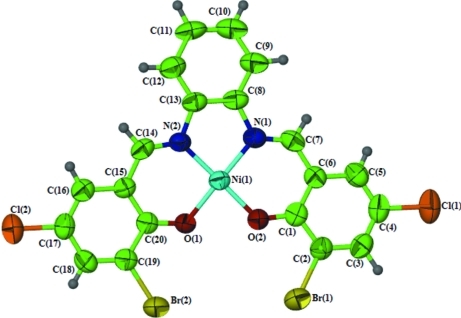
Thermal ellipsoid plot (Barbour, 2001) at the 70% probability level. Hydrogen atoms are drawn as spheres of arbitrary radius.

## supplementary crystallographic information

### Comment

Many low-spin square-planar nickel(II) complexes and high-spin octahedral nickel(II) complexes containing nitrogen and oxygen donor ligands have been reported due to their potential industrial applications (e.g. Chang *et al.*, 2008). In continuation of our study on the optical properties of nickel(II) Schiff base complexes, we report here the molecular structure of the title nickel(II) complex.

The molecular structure of the title complex is shown in Fig .1. The the Ni^II^ ion is coordinated by two phenoxy oxygen atoms and two imino nitrogen atoms in a slightly distorted square-planar geometry. The molecule is essentially planar with an rms deviation of 0.088Å for the mean plane defined by all non-hydrogen atoms in the molecule. The Ni—O bond distances [1.836 (2), 1.838 (2) Å] and Ni—N bond distances [1.853 (3), 1.858 (3) Å] are similar to those reported for related structures [1.841 (5), 1.847 (5) Å and 1.859 (6), 1.856 (6) Å, respectively, Azevedo *et al.*, 1994;  Ni-O 1.840 (5) and Ni-N 1.863 (5), 1.858 (5)Å, Wang *et al.*,  2003; Ni-O 1.839 (2) A and Ni-N 1.825 (2) Å, Niu *et al.*,  2009].

### Experimental

The Schiff base, *o*-phenylenebis(3-bromo-5-chlorosalicylidenaminate was prepared by the condensation reaction between *o*-phenylenediamine and 3-bromo-5-chlorosalicylaldehyde in ethanol. 0.1 g (0.183 mmol) of the Schiff base ligand and 0.04 g (0.183 mmol) of nickel(II) acetate tetrahydrate were dissolved in 100 ml of absolute ethanol. A few drops of triethylamine were added and the mixture was refluxed for 3 hours. After filtering, a red colored solid was obtained upon slow evaporation of the filtrate. It was recrystalised from DMF to obtain the red crystals suitable for X-ray analysis.

### Refinement

Hydrogen atoms were located in a difference Fourier map, and were allowed to refine isotropically.

### Crystal data

**Table:**

[Ni(C_20_H_10_Br_2_Cl_2_N_2_O_2_)]	*F*(000) = 1168
*M**_r_* = 599.73	*D*_x_ = 2.038 Mg m^−^^3^
Monoclinic, *P*2_1_/*c*	Mo *K*α radiation, λ = 0.71073 Å
Hall symbol: -P 2ybc	Cell parameters from 2542 reflections
*a* = 10.4289 (2) Å	θ = 2.5–22.8°
*b* = 9.2712 (2) Å	µ = 5.38 mm^−^^1^
*c* = 20.6731 (4) Å	*T* = 296 K
β = 102.101 (1)°	Tube, red
*V* = 1954.43 (7) Å^3^	0.40 × 0.10 × 0.10 mm
*Z* = 4	

### Data collection

**Table:**

Bruker APEXII CCD area-detector diffractometer	4487 independent reflections
Radiation source: fine-focus sealed tube	2921 reflections with *I* > 2σ(*I*)
graphite	*R*_int_ = 0.060
ω scans	θ_max_ = 27.5°, θ_min_ = 2.0°
Absorption correction: multi-scan (*SADABS*; Sheldrick, 1996)	*h* = −13→13
*T*_min_ = 0.222, *T*_max_ = 0.616	*k* = −12→12
18381 measured reflections	*l* = −26→25

### Refinement

**Table:**

Refinement on *F*^2^	Primary atom site location: structure-invariant direct methods
Least-squares matrix: full	Secondary atom site location: difference Fourier map
*R*[*F*^2^ > 2σ(*F*^2^)] = 0.038	Hydrogen site location: inferred from neighbouring sites
*wR*(*F*^2^) = 0.085	H atoms treated by a mixture of independent and constrained refinement
*S* = 0.99	*w* = 1/[σ^2^(*F*_o_^2^) + (0.0337*P*)^2^] where *P* = (*F*_o_^2^ + 2*F*_c_^2^)/3
4487 reflections	(Δ/σ)_max_ < 0.001
302 parameters	Δρ_max_ = 0.62 e Å^−^^3^
0 restraints	Δρ_min_ = −0.46 e Å^−^^3^

### Special details

**Table:**

Geometry. All esds (except the esd in the dihedral angle between two l.s. planes) are estimated using the full covariance matrix. The cell esds are taken into account individually in the estimation of esds in distances, angles and torsion angles; correlations between esds in cell parameters are only used when they are defined by crystal symmetry. An approximate (isotropic) treatment of cell esds is used for estimating esds involving l.s. planes.
Refinement. Refinement of *F*^2^ against ALL reflections. The weighted *R*-factor wR and goodness of fit *S* are based on *F*^2^, conventional *R*-factors *R* are based on *F*, with *F* set to zero for negative *F*^2^. The threshold expression of *F*^2^ > σ(*F*^2^) is used only for calculating *R*-factors(gt) etc. and is not relevant to the choice of reflections for refinement. *R*-factors based on *F*^2^ are statistically about twice as large as those based on *F*, and *R*- factors based on ALL data will be even larger.

### Fractional atomic coordinates and isotropic or equivalent isotropic displacement parameters (Å^2^)

**Table:**

	*x*	*y*	*z*	*U*_iso_*/*U*_eq_	
Ni1	0.42858 (4)	0.82303 (5)	0.00079 (2)	0.03697 (13)	
Br2	0.81519 (4)	0.64578 (5)	0.13971 (2)	0.05814 (14)	
Br1	0.53750 (4)	0.96193 (5)	0.22642 (2)	0.06178 (15)	
Cl2	0.88192 (10)	0.28706 (11)	−0.06471 (6)	0.0600 (3)	
Cl1	0.13100 (12)	1.35240 (12)	0.17562 (7)	0.0714 (3)	
C15	0.6015 (3)	0.5754 (4)	−0.05020 (18)	0.0378 (8)	
O2	0.4438 (2)	0.9051 (3)	0.08286 (12)	0.0459 (6)	
C16	0.6762 (4)	0.4667 (4)	−0.0737 (2)	0.0447 (9)	
N1	0.2791 (3)	0.9255 (3)	−0.03805 (15)	0.0394 (7)	
C14	0.4875 (4)	0.6290 (4)	−0.0939 (2)	0.0429 (9)	
C20	0.6425 (3)	0.6305 (4)	0.01441 (18)	0.0382 (8)	
C17	0.7884 (4)	0.4177 (4)	−0.0343 (2)	0.0442 (9)	
C1	0.3679 (4)	1.0010 (4)	0.10100 (19)	0.0418 (9)	
O1	0.5802 (2)	0.7289 (3)	0.03987 (12)	0.0418 (6)	
N2	0.4122 (3)	0.7309 (3)	−0.08005 (14)	0.0372 (7)	
C5	0.1859 (4)	1.1734 (4)	0.0825 (2)	0.0493 (10)	
C19	0.7597 (3)	0.5726 (4)	0.05273 (18)	0.0409 (9)	
C2	0.3932 (4)	1.0453 (4)	0.16778 (19)	0.0434 (9)	
C13	0.3023 (4)	0.7792 (4)	−0.12826 (18)	0.0424 (9)	
C11	0.1575 (5)	0.7925 (5)	−0.2344 (2)	0.0615 (13)	
C9	0.1218 (4)	0.9484 (5)	−0.1469 (2)	0.0548 (11)	
C10	0.0866 (5)	0.9001 (5)	−0.2110 (2)	0.0617 (12)	
C8	0.2305 (4)	0.8878 (4)	−0.10542 (18)	0.0435 (9)	
C18	0.8314 (4)	0.4705 (4)	0.0289 (2)	0.0451 (10)	
C7	0.2236 (4)	1.0247 (4)	−0.0091 (2)	0.0446 (10)	
C3	0.3200 (4)	1.1484 (4)	0.1905 (2)	0.0479 (10)	
C4	0.2165 (4)	1.2134 (4)	0.1466 (2)	0.0511 (10)	
C6	0.2602 (3)	1.0669 (4)	0.05861 (19)	0.0418 (9)	
C12	0.2644 (4)	0.7316 (5)	−0.1927 (2)	0.0559 (11)	
H14	0.471 (3)	0.581 (3)	−0.1363 (16)	0.028 (8)*	
H3	0.341 (3)	1.177 (4)	0.2365 (19)	0.052 (11)*	
H16	0.643 (3)	0.431 (4)	−0.1153 (19)	0.045 (11)*	
H18	0.901 (4)	0.432 (4)	0.0531 (19)	0.049 (12)*	
H7	0.155 (3)	1.070 (4)	−0.0322 (17)	0.039 (10)*	
H11	0.125 (5)	0.765 (5)	−0.279 (3)	0.097 (17)*	
H10	0.023 (4)	0.947 (4)	−0.239 (2)	0.057 (12)*	
H5	0.120 (3)	1.212 (4)	0.0547 (19)	0.044 (11)*	
H9	0.066 (4)	1.023 (4)	−0.132 (2)	0.069 (13)*	
H12	0.310 (4)	0.651 (5)	−0.210 (2)	0.085 (16)*	

### Atomic displacement parameters (Å^2^)

**Table:**

	*U*^11^	*U*^22^	*U*^33^	*U*^12^	*U*^13^	*U*^23^
Ni1	0.0367 (3)	0.0410 (3)	0.0324 (3)	−0.0011 (2)	0.0056 (2)	0.0021 (2)
Br2	0.0541 (3)	0.0737 (3)	0.0418 (3)	0.0081 (2)	−0.00089 (19)	−0.0011 (2)
Br1	0.0653 (3)	0.0779 (3)	0.0398 (3)	0.0091 (2)	0.0058 (2)	0.0018 (2)
Cl2	0.0570 (7)	0.0536 (6)	0.0746 (8)	0.0071 (5)	0.0256 (6)	−0.0057 (5)
Cl1	0.0824 (8)	0.0545 (7)	0.0866 (9)	0.0122 (6)	0.0392 (7)	−0.0079 (6)
C15	0.039 (2)	0.040 (2)	0.035 (2)	−0.0059 (16)	0.0088 (17)	0.0009 (16)
O2	0.0452 (15)	0.0519 (16)	0.0387 (15)	0.0094 (12)	0.0044 (12)	−0.0038 (12)
C16	0.043 (2)	0.048 (2)	0.044 (3)	−0.0076 (18)	0.010 (2)	−0.0020 (19)
N1	0.0404 (17)	0.0384 (17)	0.0381 (18)	−0.0021 (14)	0.0051 (14)	0.0054 (14)
C14	0.048 (2)	0.050 (2)	0.032 (2)	−0.0087 (19)	0.0099 (18)	−0.0027 (18)
C20	0.035 (2)	0.042 (2)	0.039 (2)	−0.0052 (16)	0.0125 (17)	0.0050 (17)
C17	0.047 (2)	0.040 (2)	0.051 (3)	−0.0036 (17)	0.023 (2)	0.0005 (18)
C1	0.046 (2)	0.036 (2)	0.046 (2)	−0.0070 (17)	0.0151 (19)	0.0036 (17)
O1	0.0373 (14)	0.0513 (15)	0.0356 (15)	0.0052 (11)	0.0048 (11)	−0.0009 (12)
N2	0.0344 (17)	0.0429 (17)	0.0332 (17)	−0.0024 (13)	0.0049 (13)	0.0026 (13)
C5	0.046 (3)	0.043 (2)	0.058 (3)	0.0019 (19)	0.010 (2)	0.006 (2)
C19	0.038 (2)	0.044 (2)	0.042 (2)	−0.0051 (17)	0.0102 (17)	0.0042 (17)
C2	0.048 (2)	0.046 (2)	0.039 (2)	−0.0016 (17)	0.0152 (18)	0.0053 (18)
C13	0.044 (2)	0.049 (2)	0.033 (2)	−0.0080 (17)	0.0054 (17)	0.0059 (17)
C11	0.067 (3)	0.076 (3)	0.034 (3)	−0.010 (2)	−0.007 (2)	0.003 (2)
C9	0.055 (3)	0.055 (3)	0.047 (3)	0.000 (2)	−0.005 (2)	0.007 (2)
C10	0.060 (3)	0.064 (3)	0.051 (3)	−0.006 (2)	−0.010 (2)	0.011 (2)
C8	0.045 (2)	0.047 (2)	0.037 (2)	−0.0087 (17)	0.0019 (18)	0.0066 (17)
C18	0.036 (2)	0.047 (2)	0.053 (3)	0.0006 (18)	0.009 (2)	0.007 (2)
C7	0.040 (2)	0.041 (2)	0.050 (3)	0.0021 (18)	0.002 (2)	0.0094 (19)
C3	0.055 (3)	0.049 (2)	0.043 (3)	−0.009 (2)	0.019 (2)	−0.004 (2)
C4	0.056 (3)	0.042 (2)	0.062 (3)	−0.0010 (18)	0.028 (2)	−0.003 (2)
C6	0.039 (2)	0.039 (2)	0.047 (2)	−0.0020 (16)	0.0074 (18)	0.0003 (17)
C12	0.059 (3)	0.065 (3)	0.041 (3)	−0.003 (2)	0.004 (2)	−0.003 (2)

### Geometric parameters (Å, °)

**Table:**

Ni1—O2	1.836 (2)	C1—C2	1.411 (5)
Ni1—O1	1.838 (2)	N2—C13	1.424 (4)
Ni1—N2	1.853 (3)	C5—C4	1.349 (6)
Ni1—N1	1.858 (3)	C5—C6	1.407 (5)
Br2—C19	1.895 (4)	C5—H5	0.87 (3)
Br1—C2	1.888 (4)	C19—C18	1.362 (5)
Cl2—C17	1.752 (4)	C2—C3	1.367 (5)
Cl1—C4	1.744 (4)	C13—C12	1.381 (5)
C15—C20	1.410 (5)	C13—C8	1.394 (5)
C15—C16	1.420 (5)	C11—C12	1.379 (6)
C15—C14	1.424 (5)	C11—C10	1.388 (7)
O2—C1	1.297 (4)	C11—H11	0.95 (5)
C16—C17	1.356 (5)	C9—C10	1.372 (6)
C16—H16	0.92 (4)	C9—C8	1.389 (5)
N1—C7	1.297 (5)	C9—H9	0.99 (4)
N1—C8	1.422 (5)	C10—H10	0.89 (4)
C14—N2	1.298 (5)	C18—H18	0.87 (4)
C14—H14	0.97 (3)	C7—C6	1.426 (5)
C20—O1	1.294 (4)	C7—H7	0.87 (3)
C20—C19	1.416 (5)	C3—C4	1.393 (6)
C17—C18	1.379 (5)	C3—H3	0.97 (4)
C1—C6	1.411 (5)	C12—H12	0.99 (4)
			
O2—Ni1—O1	83.74 (11)	C20—C19—Br2	116.8 (3)
O2—Ni1—N2	176.99 (12)	C3—C2—C1	122.4 (4)
O1—Ni1—N2	94.91 (12)	C3—C2—Br1	119.5 (3)
O2—Ni1—N1	95.14 (12)	C1—C2—Br1	118.0 (3)
O1—Ni1—N1	177.58 (12)	C12—C13—C8	119.5 (4)
N2—Ni1—N1	86.31 (13)	C12—C13—N2	126.9 (4)
C20—C15—C16	120.1 (3)	C8—C13—N2	113.6 (3)
C20—C15—C14	121.4 (3)	C12—C11—C10	119.7 (4)
C16—C15—C14	118.4 (3)	C12—C11—H11	125 (3)
C1—O2—Ni1	127.7 (2)	C10—C11—H11	115 (3)
C17—C16—C15	119.9 (4)	C10—C9—C8	118.8 (4)
C17—C16—H16	123 (2)	C10—C9—H9	118 (3)
C15—C16—H16	117 (2)	C8—C9—H9	123 (3)
C7—N1—C8	121.6 (3)	C9—C10—C11	121.1 (5)
C7—N1—Ni1	125.3 (3)	C9—C10—H10	118 (3)
C8—N1—Ni1	113.1 (2)	C11—C10—H10	120 (3)
N2—C14—C15	125.2 (4)	C9—C8—C13	120.6 (4)
N2—C14—H14	122.3 (18)	C9—C8—N1	125.6 (4)
C15—C14—H14	112.5 (18)	C13—C8—N1	113.7 (3)
O1—C20—C15	124.2 (3)	C19—C18—C17	119.7 (4)
O1—C20—C19	119.2 (3)	C19—C18—H18	122 (3)
C15—C20—C19	116.6 (3)	C17—C18—H18	118 (3)
C16—C17—C18	121.2 (4)	N1—C7—C6	126.4 (4)
C16—C17—Cl2	119.4 (3)	N1—C7—H7	118 (2)
C18—C17—Cl2	119.4 (3)	C6—C7—H7	115 (2)
O2—C1—C6	124.9 (4)	C2—C3—C4	119.4 (4)
O2—C1—C2	118.8 (3)	C2—C3—H3	120 (2)
C6—C1—C2	116.3 (3)	C4—C3—H3	120 (2)
C20—O1—Ni1	127.9 (2)	C5—C4—C3	120.9 (4)
C14—N2—C13	120.6 (3)	C5—C4—Cl1	120.5 (3)
C14—N2—Ni1	126.1 (3)	C3—C4—Cl1	118.6 (3)
C13—N2—Ni1	113.2 (2)	C5—C6—C1	120.7 (4)
C4—C5—C6	120.2 (4)	C5—C6—C7	118.9 (4)
C4—C5—H5	122 (2)	C1—C6—C7	120.3 (3)
C6—C5—H5	118 (2)	C11—C12—C13	120.2 (4)
C18—C19—C20	122.4 (4)	C11—C12—H12	118 (3)
C18—C19—Br2	120.7 (3)	C13—C12—H12	122 (3)
